# The family Carabodidae (Acari, Oribatida) VIII. The genus *Machadocepheus* (first part) *Machadocepheus leoneae* sp. n. and *Machadocepheus rachii* sp. n. from Gabon

**DOI:** 10.3897/zookeys.456.8570

**Published:** 2014-11-21

**Authors:** Nestor Fernandez, Pieter Theron, Christine Rollard, Sergio Leiva

**Affiliations:** 1 National Council of Scientific and Technological Research, Argentina (CONICET). Subtropical Biological Institut (IBS). Evolutive Genetic Laboratory FCEQ y N, Misiones National University. Felix de Azara 1552, 6°, (3300) Posadas Misiones Argentina; 2 Research Unit for Environmental Sciences and Management, North-West University, Potchefstroom Campus, 2520, South Africa; 3 Muséum National d’Histoire Naturelle, Département Systématique et Evolution, Unité OSEB, Section Arthropodes, 57 rue Cuvier. 75231, Paris cedex 05. France; 4 Fellowship, National Institute Agricultural Technology (INTA). Experimental Rural Agency, Aimogasta. 5310. La Rioja. Argentina

**Keywords:** Carabodidae, *Machadocepheus leoneae* sp. n., *Machadocepheus rachii* sp. n., Gabon

## Abstract

The genus *Machadocepheus*, being one of the more complex genera of the Carabodidae family, is briefly outlined to demonstrate this complexity. Descriptions of two new species from Gabon, *Machadocepheus leoneae* **sp. n.** and *Machadocepheus rachii* **sp. n.** are given.

## Introduction

This genus was created by Balogh in 1958 (page 20), with type species *Machadocepheus excavatus* Balogh, 1958 (page 21), but in very brief text lacking figures. Mahunka (1986) redefined the genus (page 97) and supplied very brief figures of dorsal, lateral and ventral views (Figures 42, 43, 44); the type species *Machadocepheus excavatus* was redescribed (page 125) and figures 96 (anterior view) and 97 (bothridium and sensillus) added.

The species *Machadocepheus papuanus* Balogh, 1970 (from New Guinea), was instated as the type species of the genus *Guineobodes*, erected by Mahunka 1987, and is considered by Subias (2004 updated 2014) to be a subgenus of *Pasocepheus* Aoki, 1976, as Pasocepheus (Guineobodes) (Mahunka 1987). *Machadocepheus manguiati* Corpus-Raros, 1979 was designated the type species of *Philippobodes* J & P Balogh, 1992, considered by Subias (2004, updated 2014) as synonym of *Bathocepheus* Aoki, 1978, transferring the species to *Bathocepheus manguiati* (Corpus-Raros, 1979).

*Machadocepheus foveolatus* Mahunka, 1978, was designated type species of the genus *Mauribodes* J & P. Balogh, 1992, and subsequently *Mauribodes* was considered by Subias (*op. cit*) as synonym of Diplobodes (Kalloia) Mahunka, 1985. Subias recombined *Mauribodes foveolatus* (Mahunka, 1978) as Diplobodes (Kalloia) foveolatus (Mahunka, 1978). The genus *Kalloia* was created by Mahunka 1985, with *Kalloia simpliseta* Mahunka, 1985 as type species; however at present, this species has been recombined as Machadocepheus (Kalloia) simpliseta (Mahunka, 1985).

*Machadocepheus longus* Balogh, 1962 was subsequently designated type species of *Tuberocepheus* Balogh & Mahunka, 1969, while *Machadocepheus sagitta* Balogh & Mahunka, 1966 was designated type species of the genus *Sagittabodes* J & P Balogh, 1992.

More recently, Subias (*op. cit*.) divided *Machadocepheus* into two subgenera, *Machadocepheus* and *Sagittabodes*, the first subgenus with Machadocepheus (Machadocepheus) exacavatus as type and the second with Machadocepheus (Sagittabodes) sagitta (Balogh & Mahunka, 1966) as type.

With regard to Subias’s recombination of genera and currently accepted classification of *Machadocepheus*, the changes were published and necessitate justification. We studied type material in order to not accepted. This paper specifically establishes the series of characters for the genus, and future papers will discuss other problems in terms of classification, in order to state reasons why the authors agree with some changes and disagree with others.

The genus is also complex in terms of the deposition of the type *Machadocepheus excavatus* Balogh, 1958 (see above). Balogh indicated in page 1 of his paper that “Les types des formes nouvelles que je decris ici font partie des collections du Musée Royal du “Congo Belge, a Tervuren”, without further indications, but Mahunka 1986 indicated rather confusingly in the text of the redescription (page 125) “Examined types series: Holotype and 62 paratypes.Ang.4370-1: Angola: Riv.Tchimboma, affl. E du Cuango-Muque, galerie forestière des sources. Alto Chicapa, I:VIII.1954. Station, Holotype and 30 paratypes: IRAT, 30 paratypes (1107-PO-55): HNHM, 2 paratypes: MHNG. Other material 1 specimen: Ang.16888: Angola, Environs de Dundo forêt de la Luanchino, 28.III.1962 (SANJINJE et BARROS MACHADO coll) 6 paratypes from the same sample: Holotype and 2 paratypes in the MRAT, 3 paratypes (1102-PO-85): HNHM, 1 paratype: MHNG».

First of all, the type material is not housed at the Museum Tervuren, and Mahunka never differentiated between IRAT and MRAT; MRAT most probably refers to the Musée Royal du Congo Belge Tervuren, and we suppose that the type material discussed by Balogh in 1958, and possibly that of Mahunka 1986, is housed in the latter.

Other problems with the type deposition include: Mahunka indicated: “Holotype and 62 paratypes (Holotype and 30 paratypes IRAT; 30 paratypes HNHM and 2 paratypes MHNG (total holotype, plus 62 paratypes)”; but in the last part of text indicated 6 paratypes: “Holotype and 2 paratypes MRAT, 3 paratypes HNHM and 1 paratype MHNG”. That, two holotypes are referred to, one in 1954 and another 1962, with 68 paratypes, 62 from 1954 and 6 from 1962.

We studied most species cited, except for *Machadocepheus manguiati* Corpuz-Raros, 1979, which we were unable to obtain, and *Machadocepheus longus* Balogh, 1964, which was not available on loan from HNHM. We were fortunate to later obtain large quantities of specimens (from Madagascar) in the Betsch Collection of the Muséum National d’Histoire Naturelles (MNHN), Paris, France, and were able to conduct observations using both SEM and optical microscopy. The situation *Machadocepheus longus* Balogh, 1964 will the subject of a subsequent paper.

This paper, the eighth in the series on the revision of the family Carabodidae will be structured as follows: initial studies of a series of new species, making use of SEM and optical microscopy in order to permit understanding of the structures involved. Thereafter, we aim to study type material where only optical microscopy studies are available (or possible), with the intention of clarifying the taxonomy of *Machadocepheus* and related genera.

## Material and methods

Specimens studied by means of optical microscopy were macerated in lactic acid and observed in the same medium using the open-mount technique (cavity slide and cover slip) described by Grandjean (1949) and Krantz and Walter (2009). Drawings were made using a Zeiss Axio Scope (Carl Zeiss Microscopy GmbH, Jena, Germany) compound microscope equipped with a drawing tube.

Specimens were also studied with the aid of Scanning Electron Microscopy (SEM). Specimens preserved in ethanol were carefully rinsed by sucking them into a Pasteur pipette several times, after which they were transferred to buffered glutaraldehyde (2,5 %) in Sörensen phosphate buffer (pH 7,4; 0,1 m) for two hours. After postfixation for two hours in buffered 2% OsO4 solution and being rinsed in buffer solution, all specimens were dehydrated in a series of graded ethanols and dried in a critical point apparatus. After mounting on Al-stubs with double sided sticky tape, specimens were gold coated in a sputter apparatus (Alberti and Fernandez 1988). The critical point apparatus used was an Emitech K 850 (Quorum Technologies Ltd., Ashford, Kent, United Kingdom) and the sputter a Jeol JFC-1200 (Jeol Ltd. Tokyo, Japan) (metalized 80”).

SEM observations were very complex, due to limited numbers and anatomic particularities shown by specimens. Two different types of SEM were used in order to obtain observations of adequate quality: 1) Tescan Vega II LSU (Tescan Orsay Holdings, Kohoutovice, Czech Republic) (Direction of Collections-SEM-EDS-MNHN) and 2) Hitachi SU3500 (Hitachi High-Technologies Europe, Krefeld, Germany) (Plateau technique de Microscopie Electronique et de Microanalyse (PMEM) (MNHN) using accelerating voltage of 15 Kv and 10 Kv respectively.

In the legends to Figures, images obtained with Tescan Vega II LSU are indicated with a small number 1 and those obtained with Hitachi SU3500, with a small number 2.

Measurements taken: total length (tip of rostrum to posterior edge of notogaster); width (widest part of notogaster) in micrometers (μm).

Leg chaetotaxy studies executed with the aid of standard, polarized and phase contrast microscopes are provisional, due to the fact that only adult specimens were available for study. Setal formulae of the legs include the number of solenidia (in parentheses); tarsal setal formulae include the famulus (ε).

### Morphological terminology and abbreviations

Morphological terms and abbreviations used are those developed by F. Grandjean (1928–1974) (*cf.* Travé and Vachon 1975; Norton & Behan-Pelletier (in Krantz and Walter 2009); Fernandez et al. 2013; Fernandez et al. 2013a, b; Fernandez et al. 2013. For setal types Evans 1992: 73; and for ornamentation of cuticular surfaces Murley 1951 (in Evans *op. cit*: 9) were used.

### Institutions

MNHN: Muséum National d’Histoire Naturelle, Paris, France; MNHG: Museum Natural History Geneva; HNHM: Hungarian Natural History Museum; MRAT: probably Musée Royal du Congo Belge Tervuren; IRAT: unknown.

## New taxa descriptions

### Machadocepheus leoneae sp. n.

Taxon classificationAnimaliaOribatidaCarabodidae

http://zoobank.org/FAF67C3C-7615-451F-93E2-F7720CBA5597

Figures 1 –37

#### Etymology.

The specific epithet is dedicated in homage to Mrs. Leone Hudson, our efficient and helpful collaborator who enormously facilitated our work.

#### Material examined.

Holotype and four paratype females. Holotype ♀ Makokou, northeastern province of Ogoové-Ivindo, 500 m alt. dense evergreen humid forest, I.1974, Y. Coineau, deposited in MNHN (Muséum National d’Histoire Naturelle, Paris).

Paratypes. Same data as holotype, 4 ♀ (2 in MNHN; 2 in MNHG). All specimens are preserved in 70% ethanol.

Type locality. Makokou, province of Ogoové-Ivindo, northeastern Gabon; situated at 0°34'0"N, 12°52'0"E. Material used for SEM observations not deposited.

#### Diagnosis adult female.

Elongate animals; *ro*, *in*, notogastral, sub-capitular, epimeral, genital, aggenital, adanal, anal setae, simple; *le*, lanceolate, barbate. Prodorsum truncate pyramid shape; elevated interlamellar process, divided sagittally by a deep furrow into two promontories; *in* setae situated anteriorly, directing posteriorly. Deep posterior prodorsal depression. Sensillus uncinate, curving upward; bothridial ring and bothridial tooth present; *ro* setae curving, directing medially; *le* setae situated ventrally on lamellar apical zone. Lamellae lacking lamellar tip; lamellar furrow with deeper medial structure; superior cornea of naso convex elevation. Notogaster characteristic: notogastral anterior depression with three anterior transversally aligned parallel cuticular folds; posterior zone with two large cavities, separated by longitudinal ridge, terminating in *c_1_* setae, which are positioned on triangular convexity. Elevated medial notogastral zone with three pairs of aligned medial promontories with *da*, *dm*, *dp* setae and lateral semicircular promontories that bear *la*, *lm*, *lp*, *h_1_*, *h_2_*, setae. Behind elevated zone, posterior notogastral depression slightly concave; near circumgastric depression, a more or less flat zone with small protuberances present.

Notogastral setae, fifteen pairs (holotrichy unideficient): *c_1_*, *c_2_*, *c_3_*, *da*, *dm*, *dp*, *la*, *lm*, *lp*, *h_1_*, *h_2_*, *h_3_*, *p_1_*, *p_2_*, *p_3_*.

Supratutorial depression with three pocket depressions, one internal, another anterior and a third posterior to supratutorial depression. Bothridia cup-shaped with smooth bothridial ring and bothridial tooth. Lyrifissures *ih*, *ips* present. Subcapitular setae *h* on large promontories. Epimere 1 with two promontories; epimere 2, one promontory; epimere 3 two promontories; epimere 4 two promontories. Epimeral chaetotaxy 3-1-3-3; anterior aggenital furrow present. Genital fig small in relation to anal fig; four pairs of genital setae; two pairs of anal setae; aggenital and adanal setae similar in length and shape; lyrifissures *iad* well discernible between *ad_3_* and *ad_2_*. Several large and small depressions visible on lateral anal fig.

#### Description.

***Measurements*.** SEM: 501 μm (515–424) × 310 μm (327–295) (measurements on four specimens). Light microscopy: 512 μm (519–443) × 318 μm (338–301) (measurements on five specimens).

**Figures 1–5.**
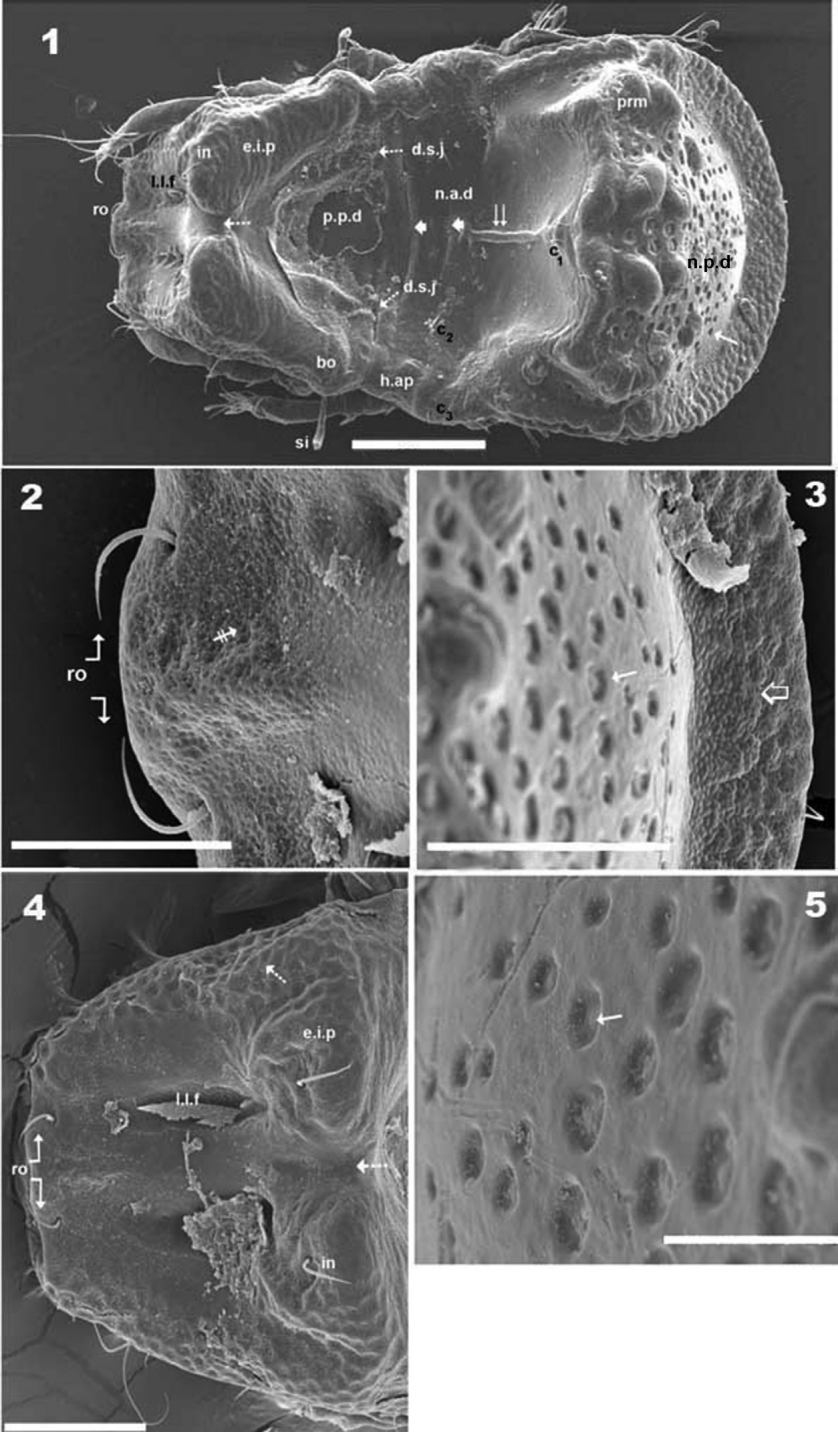
*Machadocepheus leoneae* sp. n., adult female. SEM observations. **1** dorsal view (1) **2** anterior zone of prodorsum, dorsal view (1) **3** posterior notogastral zone, dorsal view (1) **4** prodorsum, dorsal view (1) **5** fovea, posterior notogastral zone, dorsal view (2). Abbreviations: see “Material and methods”. Scale bar: **1** = 100 μm; **2** = 30 μm; **3–4** = 50 μm; **5** = 20 μm.

***Shape*.** Elongate ovoid (Figures 1, 7).

**Figures 6–8.**
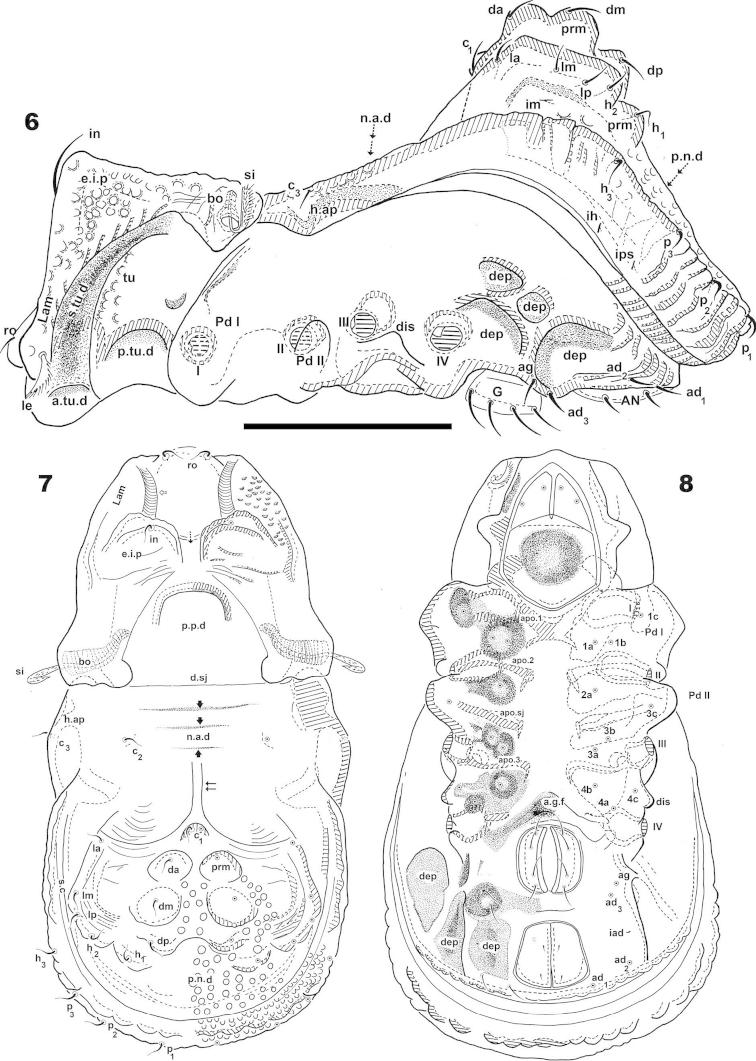
*Machadocepheus leoneae* sp. n., adult female. Optical observations. **6** lateral view **7** dorsal view **8** ventral view. Abbreviations: see “Material and methods”. Scale bar: **6–8** = 145 μm.

***Colour*.** Specimens without cerotegument, light to dark brown, observed in reflected light.

***Cerotegument*.** Thin layer (0.8–1.7 μm) covering entire body and legs (Figures 15, 32 indicated by ⇪), permitting observation only of the more prominent cuticular microsculpture (Figures 13, 25, 32). When removed, detailed microsculpture becomes visible (Figures 16, 32), however complete removal was necessary for optical microscopy.

***Integument*.** Two sizes of ornamentations: *Small*: 0.7–1.7 μm: 1) slightly foveate distributed throughout body (except notogastral zone near circumgastric depression *s.c*) (Figures 2, 13, 15, 17, 18 indicated by ⇞); 2) small protuberances, notogastral zone near *s.c* (Figures 3, 27, 30 indicated by ⇪). *Large*: 5–10 μm. Foveate, two types: 1) simple rounded fovea, situated in the elevated zone of notogaster (Figures 1, 3, 5, 9, 27, 30, 31, 32 indicated by ↑); 2) polyhedral fovea (distributed side by side), situated on prodorsum, lateral notogastral zone, and near *la* setae (Figures 4, 9, 10, 13, 25, 28 indicated by ↓).

**Figures 9–10.**
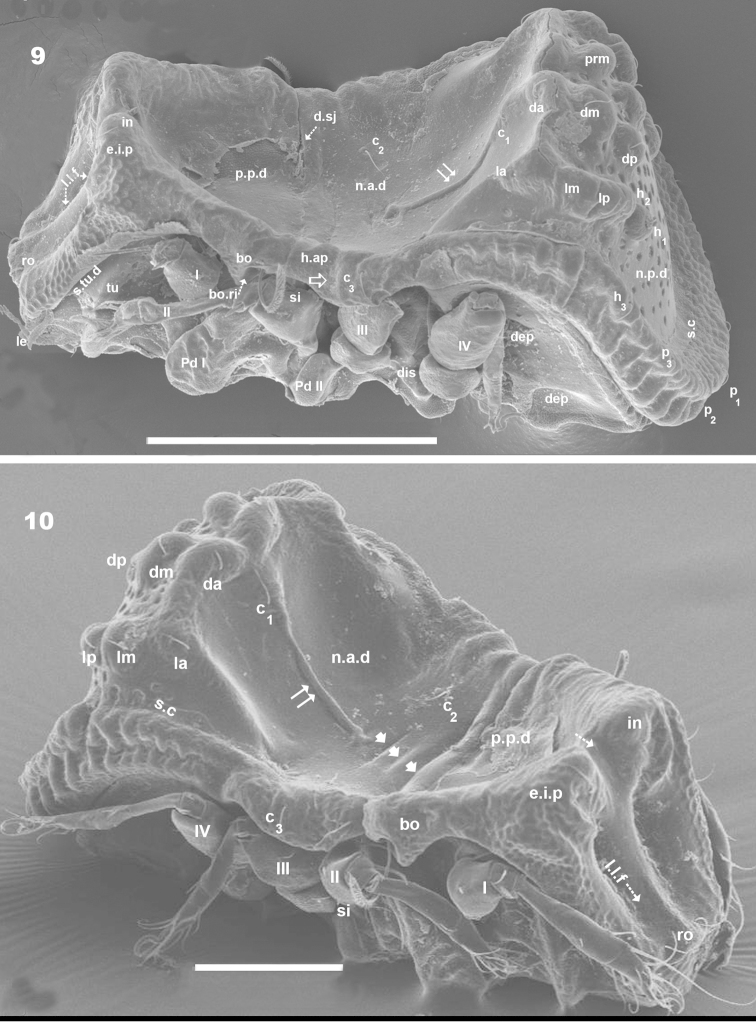
*Machadocepheus leoneae* sp. n., adult female. SEM observations. **9** dorsal inclined view (2) **10** dorsal anteroposterior view (1). Abbreviations: see “Material and methods”. Scale bars: **9** = 200 μm; **10** = 100 μm.

***Setation*.** Setae *ro*, *in*, notogastral, sub-capitular, epimeral, genital, aggenital, adanal, anal: simple (Figures 4, 6, 7, 14, 17, 18, 19, 20, 21, 23, 24, 25, 28, 32); *le*, lanceolate, barbate (Figure 16, 21, 25).

***Prodorsum*.** Shape: Truncate pyramid (Figure 6, 9, 10); truncate triangle in dorsal view (Figure 1, 4, 7); truncate inverted triangle in frontal view (Figures 19, 28).

Large elevated interlamellar process (*e.i.p*) (Figures 6, 9, 10, 12), large deep furrow dividing *e.i.p* sagittally into two promontories (Figures 6, 9, 10, 19, 27, 28, indicated by ⇡). Posterior prodorsal zone (*p.p.d*) deeply depressed (Figures 1, 7, 9, 10, 27, 29); depression continuous with notogastral anterior depression (*n.a.d*); dorsosejugal furrow (*d.sj*) (Figures 1, 7, 9, 10, 29) evidently separating *p.p.d* and *n.a.d*. Three pairs of setae; size *in*> *le*> *ro* (Figures 6, 19, 21). Sensillus uncinate, curving upward (Figure 13), bothridial ring (*bo.ri*) and bothridial tooth (*bo.to*) present.

**Figures 11–12.**
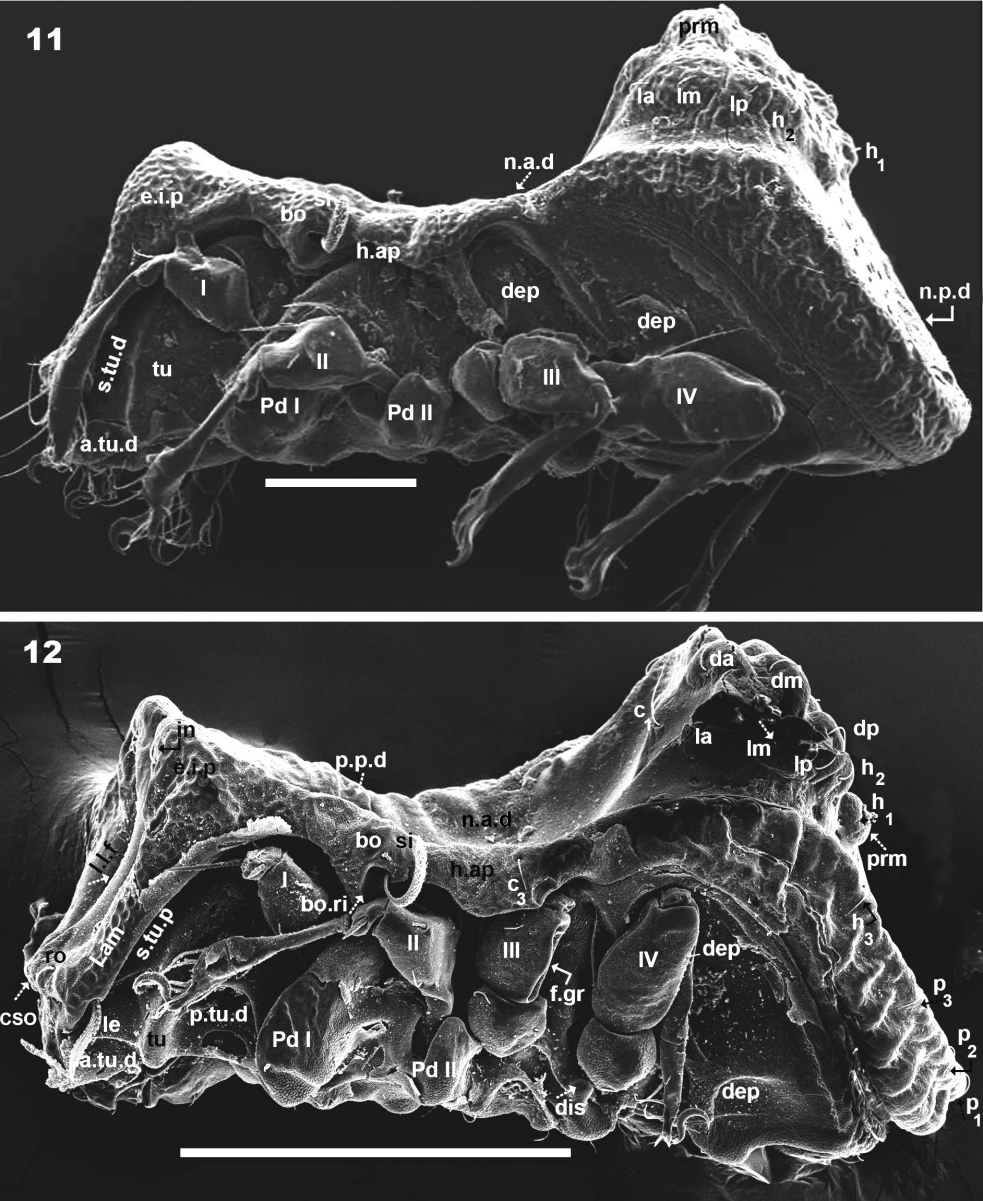
*Machadocepheus leoneae* sp. n., adult female. SEM observations. **11** lateral view **12** inclined lateral view. Abbreviations: see “Material and methods”. Scale bar: **11** = 100 μm; **12** = 200 μm.

**Figures 13–18.**
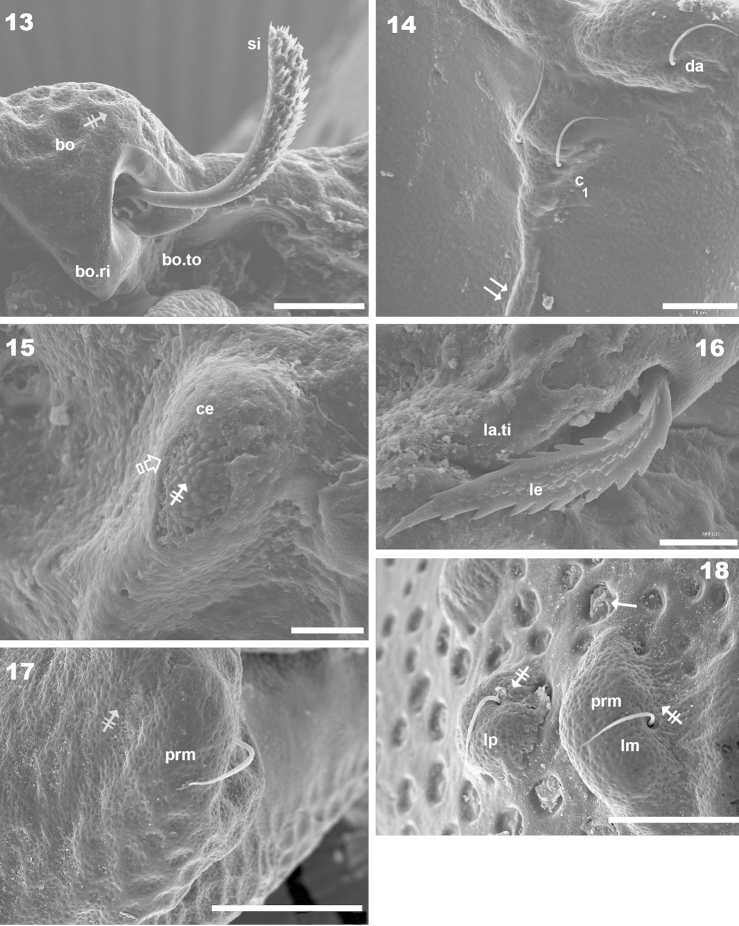
*Machadocepheus leoneae* sp. n., adult female. SEM observations. **13** bothridium and sensillus, lateral view (1) **14** posterior zone of notogastral anterior depression (2) **15** promontories with and without cerotegumental layer (2) **16** lamellae anterior zone, lateral view (2) **17** promontories with dorso-central setae (1) **18** lateral promontories with *lm*, *lp*, setae (1). Abbreviations: see “Material and methods”. Scale bar: **13–14** = 20 μm; **15–16** = 10 μm; **17–18** = 30 μm.

Setae *ro* inserted slightly anteriorly or at level of *le* insertion (Figures 19, 21); curving, directing interiorly; apical tips not touching each other (Figures 2, 4); *in* setae inserted on anterior zone of *e.i.p* promontories, curving, directing backward, paraxial to medial plane; inserted slightly externally to *ro* insertion level (Figures 1, 4, 6, 7, 10, 19, 28); *le* setae situated ventrally on lamellar apical zone (Figures 6, 16, 19, 21, 25, 26).

**Figures 19–21.**
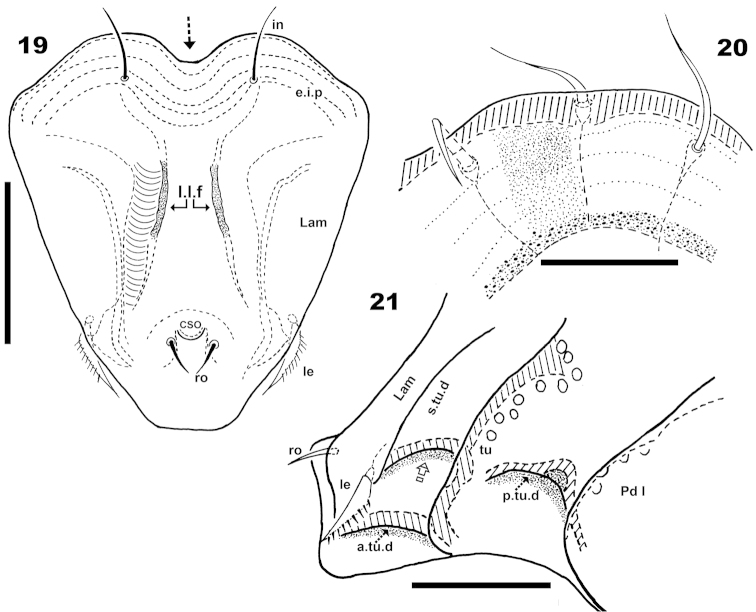
*Machadocepheus leoneae* sp. n., adult female. Optical observations. **19** frontal view **20** Promontories, lateral view **21** prodorsum anterior zone, lateral view. Abbreviations: see “Material and methods”. Scale bar: **19–21** = 100 μm.

**Figures 22–26.**
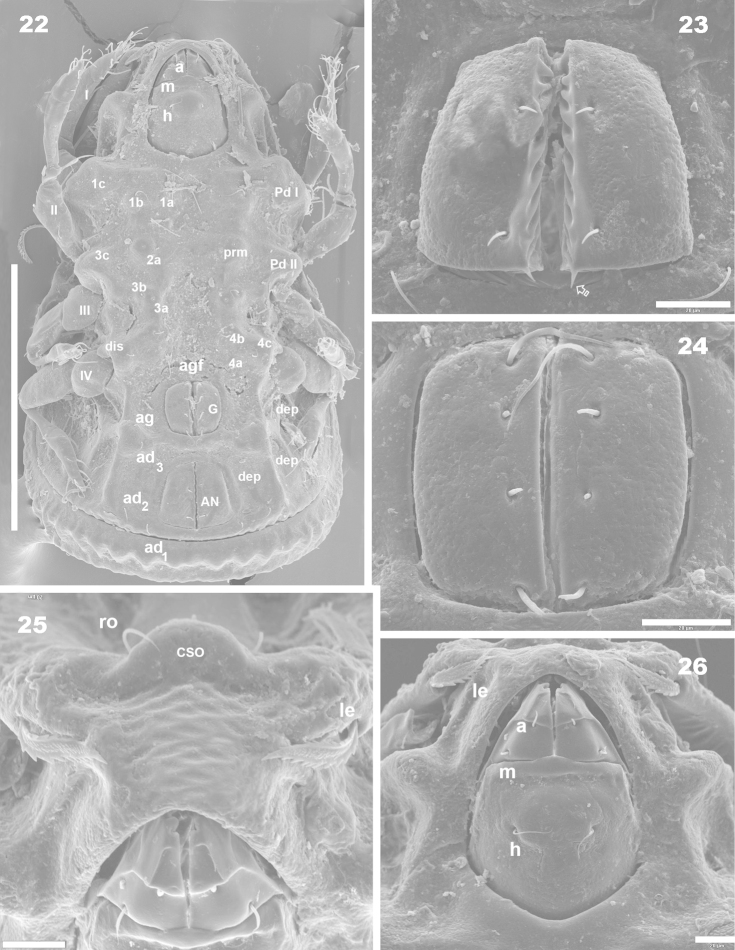
*Machadocepheus leoneae* sp. n., adult female. Optical observations. **22** ventral view (2) **23** anal fig (1) **24** genital fig (1) **25** aspis frontal view (2) **26** infracapitulum and surrounding zone (1). Abbreviations: see “Material and methods”. Scale bar: **22** = 200 μm; **23–26** = 20 μm.

Rostral margin slightly rectangular to hexagonal (Figures 19, 28). Lamellae run dorso laterally, without lamellar tips (Figures 16, 21, 25, 26); *le* setae inserted ventrally (Figure 25); inner paraxial margin of lamellae demarcated by large deep furrow (*l.l.f*) (Figures 9, 10, 12, 19, 28). In frontal view (Figures 19, 28) *l.l.f* showing deeper medial zone. The superior cornea of naso (*cso*) clearly visible as convex elevation situated at more or less same level as *ro* setal insertion (Figures 19, 25).

**Figures 27–32.**
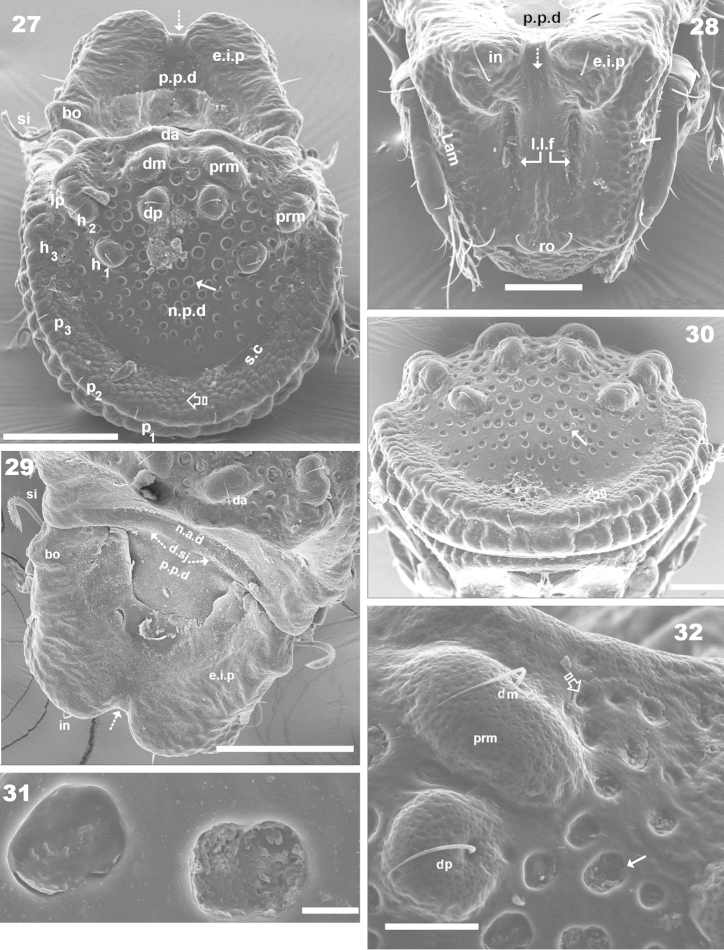
*Machadocepheus leoneae* sp. n., adult female. Optical observations. **27** posterior general view (1) **28** fontal view (1) **29** prodorsum and anterior notogastral zone, posterior view (2) **30** notogastral posterior view (2) **31** notogastral ornamentation, rounded fovea (2) **32** promontories with *dm*, *dp* setae (1). Abbreviations: see “Material and methods”. Scale bar: **27–29** = 100 μm; **28–32** = 50 μm.

***Notogaster*.** Shape: in dorsal view anterior part rectangular and posterior part oval (Figures 1, 7); in lateral view, anterior part clearly concave in medial zone and rectilinear exteriorly, rest convex with irregular promontories (Figures 6, 9, 10, 11, 12); *d.sj* narrow, rectilinear, well delimited (Figures 1, 7, 9); notogastral anterior depression (*n.a.d*) ovoid and conspicuous. Fifteen pairs (holotrichous, unideficient) of notogastral setae; *c_1_*, *c_2_*, *c_3_*, *da*, *dm*, *dp*, *la*, *lm*, *lp*, *h_1_*, *h_2_*, *h_3_*, *p_1_*, *p_2_*, *p_3_*.

The notogaster has: anterior depression (*n.a.d*) occupying anterior notogastral zone; elevated zone situated in medial to posterior part of notogaster; posterior to elevated zone, slightly concave notogastral posterior depression (*n.p.d*) (Figures 1, 6, 7, 9, 27, 11), terminating in more or less flat slightly inclined zone with small protuberances (Figure 3 indicated by ➮); Circumgastric depression (*s.c*) present anterior to zone of small protuberances (Figures 7, 9, 27).

Complex *n.a.d*, three transversally aligned parallel cuticular folds situated posterior to *d.sj* (Figures 1, 7, 10 indicated by ➧). In posterior zone, two large concavities, separated by longitudinal ridge (Figures 1, 7, 9, 10, 14 indicated by ⇈). Ridge terminating in triangular shape, situated near first pair of protuberances on elevated zone bearing *da* setae. Triangular zone of cord termination bearing *c_1_* setae (Figures 1, 7, 10, 14).

Elevated zone presenting a series of aligned medial promontories (three pairs, variably developed) bearing setae *da*, *dm*, *dp*; and lateral semi-circular promontories bearing setae *la*, *lm*, *lp*, *h_1_*, *h_2_*. Setae *c_3_* situated on humeral apophysis (*h.ap*), *c_2_* laterally situated near *h.ap*, but in the depression on *n.a.d* (Figures 1, 7, 9, 10, 27, 32). Four pairs of setae, *h_3_*, *p_1_*, *p_2_*, *p_3_* situated marginally.

Humeral apophysis (*h.ap*) very long, clearly visible as a pronounced projection, giving characteristic shape to anterior zone of notogaster (Figures 6, 9, 10, 11, 12).

***Lateral region*** (Figures 6, 9, 10, 11, 12). Lamellae (*lam*) easily discernible, large, without sharp *la.ti*, and with rounded elevated zone at level of *le* insertion (Figure 16, 21).

Tutorium (*tu*): rod-like curving ridge, clearly visible (Figures 11, 12). Between lamellae and tutorium, deep supratutorial depression (*s.tu.d*) running parallel to both structures; pocket depression (*a.tu.d*) anteriorly and posterior pocket depression (*p.tu.d*) present; small depressions posterior to *p.tu.d* as well as others situated on the interior of *s.tu.d* (Figure 21 indicated by ⇪).

Bothridia cup-shaped with smooth bothridial ring (*bo.ri*); *bo.ri* incomplete, with bothridial tooth (*bo.to*) clearly discernible (Figures 6, 9, 10, 11, 12, 13). Sensillus uncinate, arching apex (Figure 13). Pedotectum I: prominent extended lamina covering first acetabulum, rounded apex. Pedotectum II: small ovoid lamina (Figures 6, 9). Humeral apophysis (*h.ap*) long, extended structure, rounded apex, basally curved; anterior tip overlapping posterior of bothridium (Figures 11, 12).

Notogastral promontories bearing setae clearly discernible (Figures 11, 12, 17, 18); promontories show several internal layers as in Figure 20.

Only lyrifissures *ih* and *ips* clearly visible. Discidium easily discernible as triangular structure with rounded apex. Several large depressions (*dep*) clearly discernible behind acetabulum IV (Figures 6, 9).

***Ventral region*.** Infracapitulum with setae *h*, *m*, *a* clearly visible; setae *h* situated on large promontories (Figure 29). Epimere slightly elevated, delimited by shallow furrow (*bo.1*, *bo.2*, *bo.sj*). Epimere1 with two well delimited promontories, bearing setae *1a*, *1b*; epimere 2 only one promontory, bearing setae *2a*; epimere 3 with two promontories, bearing setae *3a* and *3b*; epimere 4 bearing two promontories with setae *4a* and *4b* (Sidorchuk and Norton 2010). Apodemes (*apo.1*, *apo.2*, *apo.3* and *apo.4*) clearly discernible (Figures 8, 22). Epimeral chaetotaxy 3-1-3-3; Pd I, Pd II and *dis* easily discernible; aggenital furrow *a.g.f* clearly visible, situated anteriorly to genital fig. Genital fig small relative to anal fig (Figure 22); four pairs of long genital setae (Figure 24); anal fig with two pairs of setae; one pair situated anteriorly and the other posteriorly, both setae small, but well discernible; fig terminating in small sharp tip (Figure 23). Aggenital and adanal setae similar, long, simple; *ag* and *ad_3_*, situated on promontory; *ad_2_*, *ad_1_*, situated laterally at level of posterior tip of anal fig (Figure 8, 22). Lyrifissures *iad* clearly discernible, situated laterally between *ad_3_* and *ad_2_* outside *dep*. Laterally to anal fig and marginally to ventral shield, several large and small depressions (Figures 8, 22).

***Posterior view*.** This view is very important, permitting clarification of several interesting aspects such as: a) the cuticular microsculpture and the *n. p.d* (Figure 27); b) the *p.p.d* and its relation to the *n.a.d*, as well as the related position of *d.sj*. (Figure 29); c) the relative positions of *e.i.p* and *p.p.d* (Figure 27); d) related position of central and lateral notogastral promontories (Figures 27, 30); e) disposition of *s.c* (Figure 27) and f) shape and distribution of setae and promontories (Figure 32).

**Figures 33–37.**
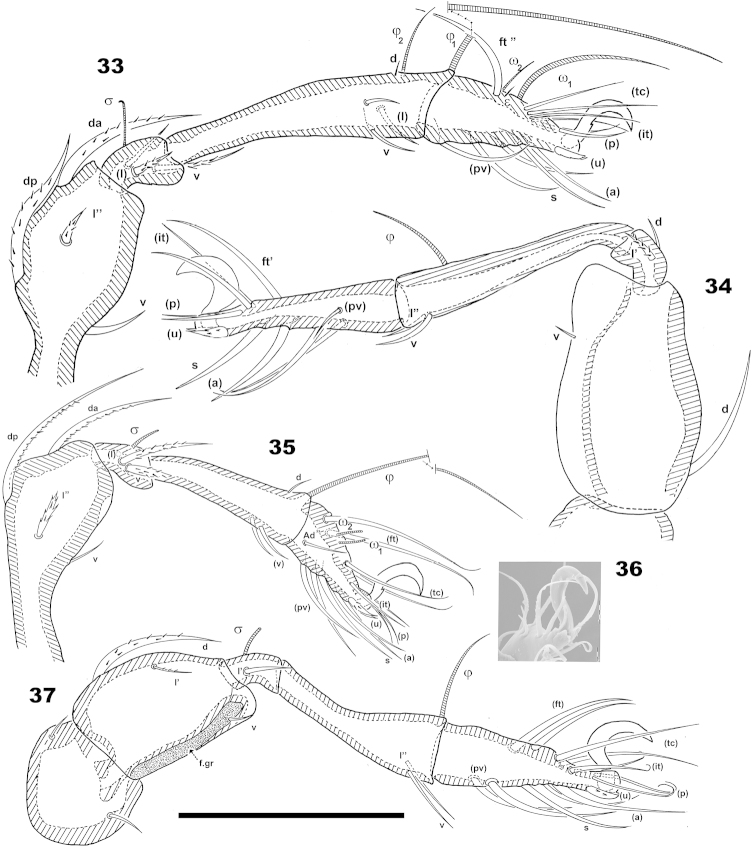
*Machadocepheus leoneae* sp. n., adult female. Optical observations. **33** leg I antiaxial view **34** leg IV antiaxial view **35** leg II antiaxial view **36** apical zone, leg II(1) **37** leg III, antiaxial view. Abbreviations: see “Material and methods”. Scale bars: **35–37** = 50 μm; **36** = 10 μm.

***Legs*** (Figures 33–37). All legs monodactyle. Setal formulae I (1-4-3-4-15-1) (1-2-2); II (1-4-3-3-16-1) (1-1-2); III (2-3-1-2-15-1) (1-1-0); IV (1-2-1-2-12-1) (0-1-0). Figure 36 showing shape of anterior setae, tarsus II. Observation of the shape of especially (*u*), (*p*), difficult in optical observations. Setae *ft*` absent from tarsus I, but present on tarsus II in all specimens studied.

Tibia I: solenidion φ_1_ on small apophysis; tibia I, II, setae *d* present, situated near solenidion. Femur IV presenting a conspicuous ventral carina.

### Machadocepheus rachii sp. n.

Taxon classificationAnimaliaOribatidaCarabodidae

http://zoobank.org/6787E360-4484-44A2-8DAD-5A73F7D7E633

Figures 38 –72

#### Etymology.

The specific epithet is dedicated in homage to Mr Rachid Kebir of Muséum National d’Histoire Naturelles, Paris, who assisted us with great kindness and friendship on many occasions over the past 20 years.

#### Material examined.

Holotype and four Paratype females. Makokou, northeastern province of Ogoové-Ivindo, 500 m. alt.dense evergreen humid forest, I.1974, Y. Coineau, deposited in MNHN. Paratypes. Same data as holotype, 4 ♀ (2 in MNHN; 2 in MNHG). All specimens preserved in 70% ethanol. Type locality. Makokou, province of Ogoové-Ivindo, northeastern Gabon; situated at 0°34'0"N, 12°52'0"E. Material used for SEM observations not deposited.

#### Diagnosis adult female.

Thin cerotegumental layer covering entire body, giving the impression of a smooth surface. Setae *ro*, *in*, notogastral, sub-capitular, epimeral, genital, aggenital, adanal, anal, simple sharply tipped; *le* lanceolate, barbate.

Polyhedral prodorsum; interlamellar process elevated, divided sagittally by large deep furrow; *in* setae situated anteriorly, directing posteriorly. Conspicuous deep posterior prodorsal depression present. Bothridium cup-shaped; bothridial ring and bothridial tooth present. Sensillus uncinate, upturned; *le* setae situated ventrally on apical zone of lamellae. Lamellae running dorsolaterally, lacking lamellar tip; large, deep, shallow lamellar furrow demarcating paraxial lamellar margin. Superior cornea of naso clearly visible as convex elevation situated anterior to insertion level of *ro* setae.

Anterior part of notogaster rectangular; posterior part oval with some irregularities and less conspicuous promontories, dorsosejugal furrow narrow, rectilinear, hardly discernible. Fifteen pairs of notogastral setae (holotrichy unideficient), *c_1_*, *c_2_*, *c_3_*, *da*, *dm*, *dp*, *la*, *lm*, *lp*, *h_1_*, *h_2_*, *h_3_*, *p_1_*, *p_2_*, *p_3_*. Notogaster presenting: notogastral anterior depression; elevated zone; slightly concave posterior depression. Notogastral anterior depression simple, with transversally aligned parallel cuticular folds. Elevated zone with three pairs of poorly developed promontories that bear *da*, *dm*, *dp* setae; and lateral semicircular, poorly developed promontories, that bear *la*, *lm*, *lp*, *h_1_*, *h_2_* setae. Humeral apophysis long, clearly visible.

Tutorium: rod-like curving cuticular thickening; supratutorial depression present; along with three pocket-shaped depressions, one anterior tutorial depression, one posterior tutorial depression and a small depression situated internally to supratutorial depression. Pedotecta I, prominent extended lamina, rounded apex; Pedotecta II small, ovoid lamina. Lyrifissures *ih*, *ips* clearly visible. Discidium: polyhedral structure with rounded apex. Depressions behind acetabulum IV; one of them elongated, concealing tarsus during folding legs process. Series of aligned depressions in medial zone. Epimeral chaetotaxy 3–1-3–3; anterior genital furrow clearly visible; four pairs of long genital setae; two pairs of small anal setae; anal fig terminating in small sharp tip; aggenital and adanal setae similar length; lyrifissures *iad* not discernible.

#### Description.

***Measurements*.** Light microscopy: 421 μm (396–426) × 262 μm (238–268) (on six specimens). SEM microscopy: 416 μm (398–416) × 176 μm (173–181) (on six specimens, not deposited).

**Figures 38–40.**
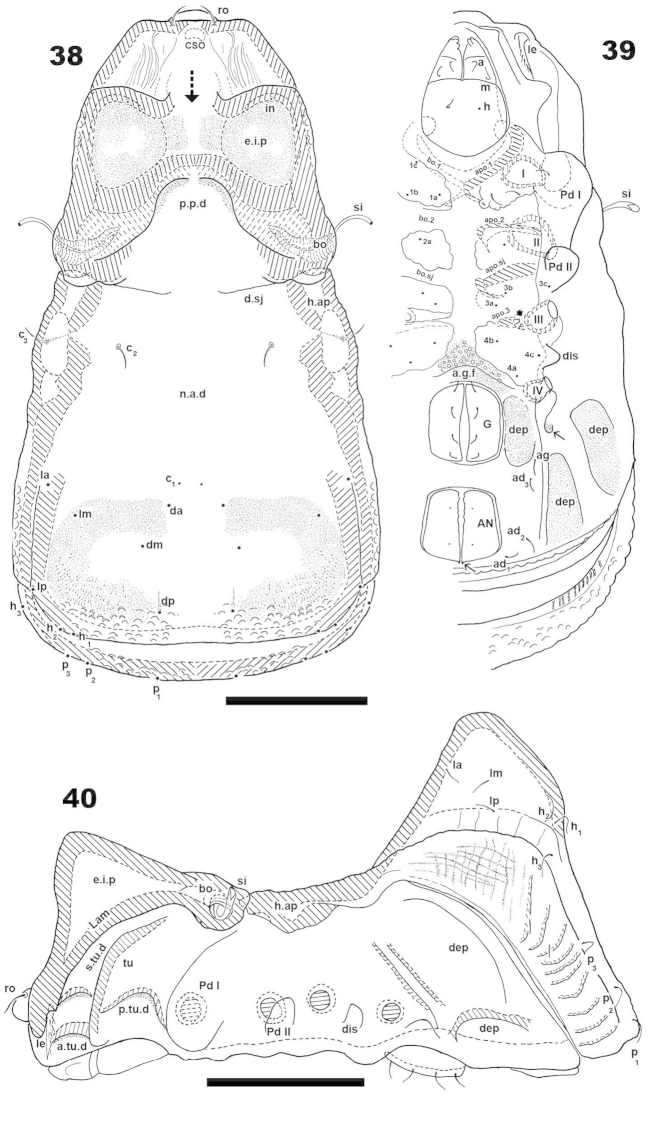
*Machadocepheus rachii* sp. n., adult female.Optical observations. **38** dorsal view **39** ventral view **40** lateral view. Abbreviations: see “Material and methods”. Scale bar: **38–40** = 90 μm.

**Figures 41–45.**
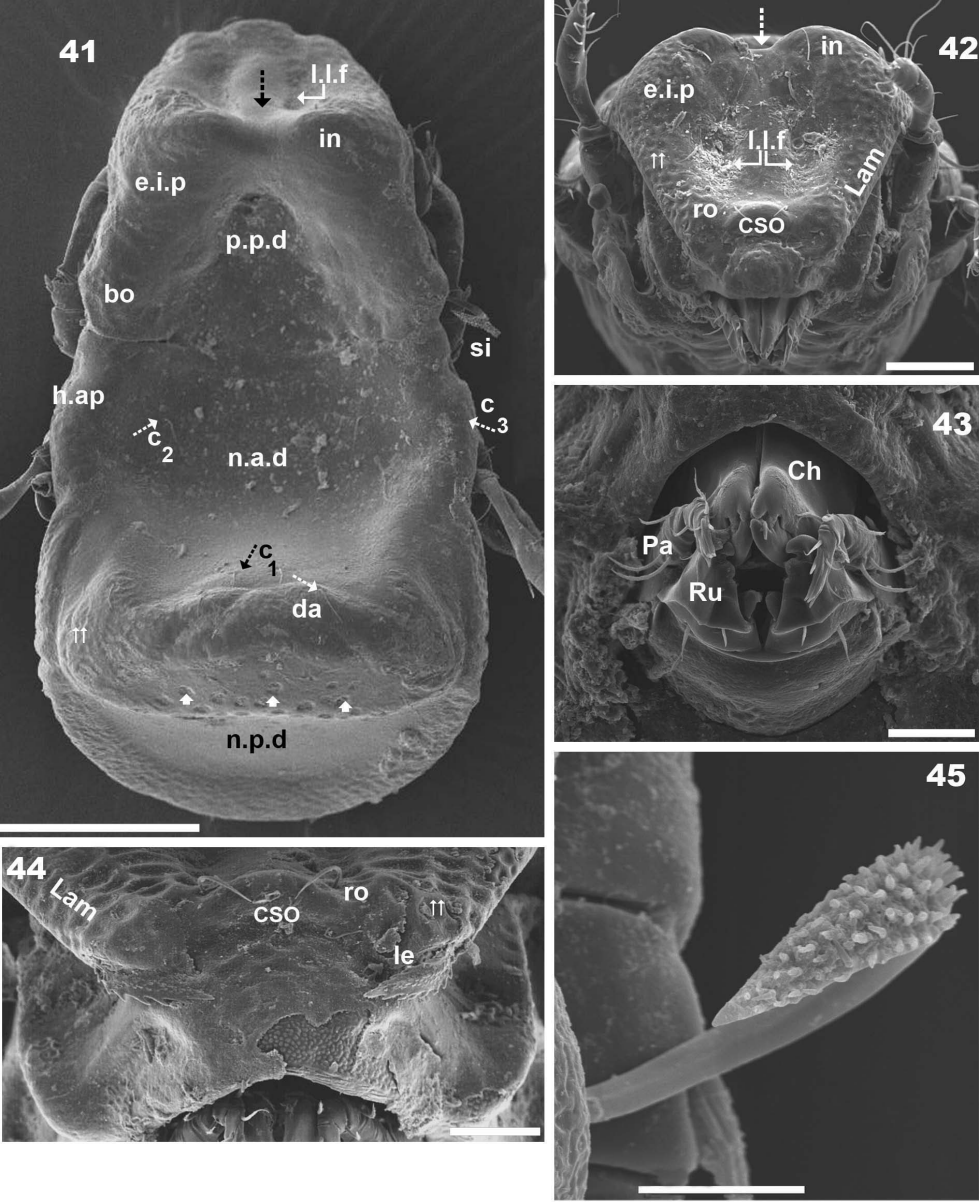
*Machadocepheus rachii* sp. n., adult female. SEM observations. **41** dorsal view (2) **42** prodorsum, frontal view (1) **43** gnathosoma, frontal view (1) **44** aspis, frontal view (1) **45** sensillus (1). Abbreviations: see “Material and methods”. Scale bar: **41** = 100 μm; **42** = 50 μm; **43–44** = 20 μm; **45** = 10 μm.

***Shape*.** Ovoid (Figures 38, 41).

***Colour*.** Specimens without cerotegument, light to dark brown, when observed in reflected light.

***Cerotegument*.** Thin layer 1.5 μm (1.3–2.5) covering the entire body and legs (Figures 38, 41, 42, 44, 47, 48, 49 indicated by ⇡, 52, 53, 56, 57, 58, 59, 63), permitting observation of only large cuticular microsculptures (Figures 44, 46, 48, 53, 58, 59, 62), giving the impression of a smooth surface. Complete removal was necessary for optical microscopy, once removed, detailed microsculpture became visible (Figure 63).

**Figures 46–47.**
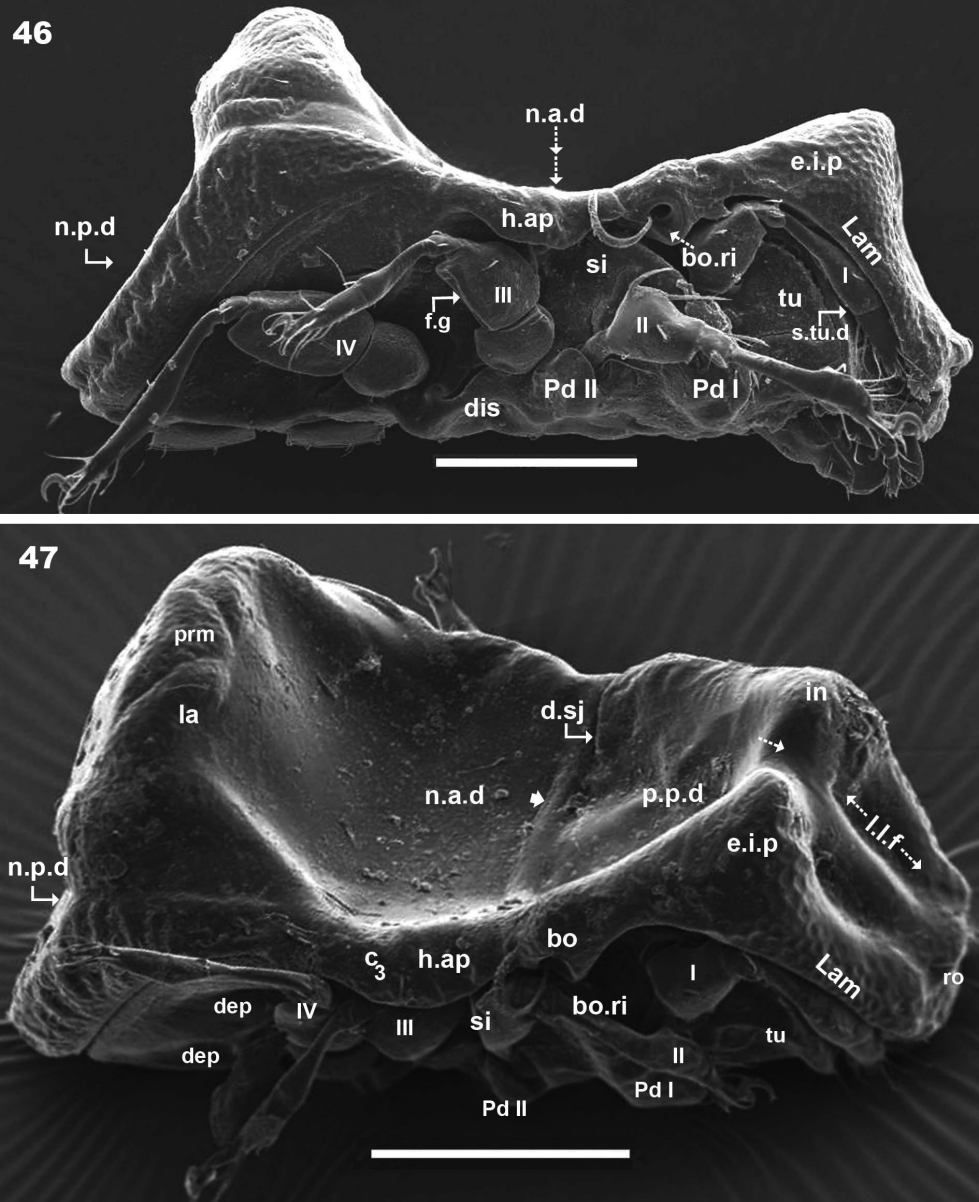
*Machadocepheus rachii* sp. n., adult female. SEM observations. **46** lateral view (2) **47** inclined lateral view (1). Abbreviations: see “Material and methods”. Scale bar: **46–47** = 100 μm.

**Figures 48–53.**
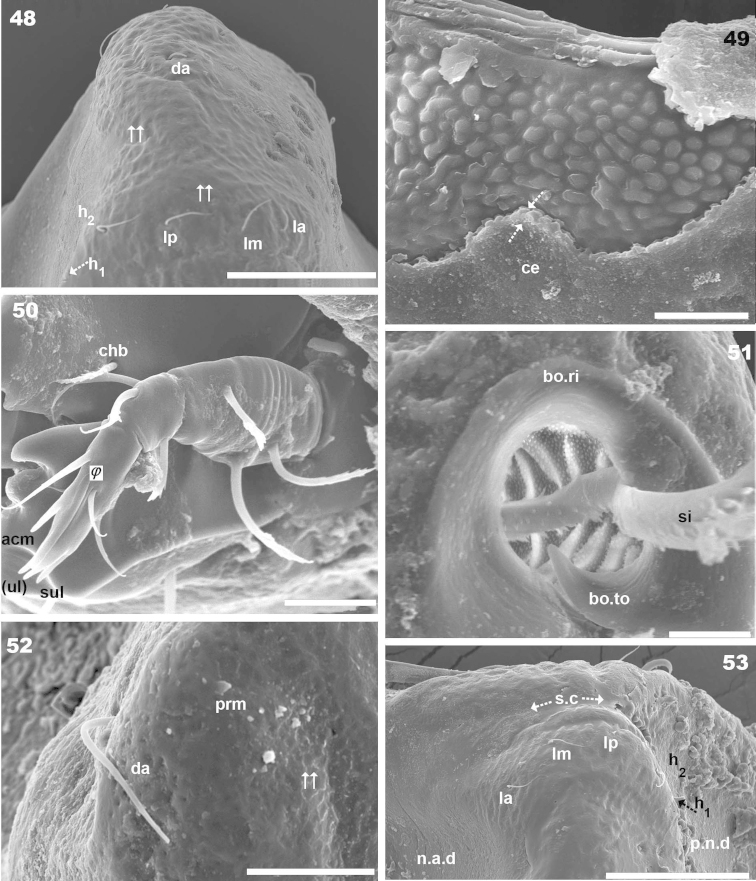
*Machadocepheus rachii* sp. n., adult female. SEM observations. **48** elevated notogastral zone (1) **49** tegument (1) **50** palp (1) **51** bothridia (1) **52** promontory with *da* setae (1) **53** elevated lateral notogastral zone (1). Abbreviations: see “Material and methods”. Scale bar: **48** = 40 μm; **49, 51** = 5 μm; **50, 52** = 10 μm; **52; 53** = 50 μm.

***Integument*.** Two sizes of ornamentations: *Small* 1.2–3.5 μm, 1) small ovoid to irregular protuberances, distributed throughout prodorsum and notogaster (except notogastral zone near *s.c*) (Figure 49) 2) irregular elongate protuberances, notogastral zone near *s.c* (Figures 41, 58, 61 indicated by ⇪). *Large* 7.2–7.9 μm, two types: 1) simple rounded fovea (Figure 63), situated on posterior part of elevated zone of notogaster (Figures 41, 58, 59, 62 indicated by ➧); 2) polyhedral fovea (distributed side by side), situated on prodorsum (*e.i.p*, lamellae, near *ro* insertion, bothridium), notogaster (elevated zone, lateral zone) (Figures 41, 42, 48, indicated by ⇈).

***Setation*.** Setae *ro*, *in*, notogastral, subcapitular, epimeral, genital, aggenital, adanal, anal: simple, sharply tipped (Figure 60) (Figures 38, 39, 40, 42, 44, 48, 52, 53, 57, 58, 59, 62, 51); *le* lanceolate, barbate (Figures 44, 55, 56).

**Figures 54–57.**
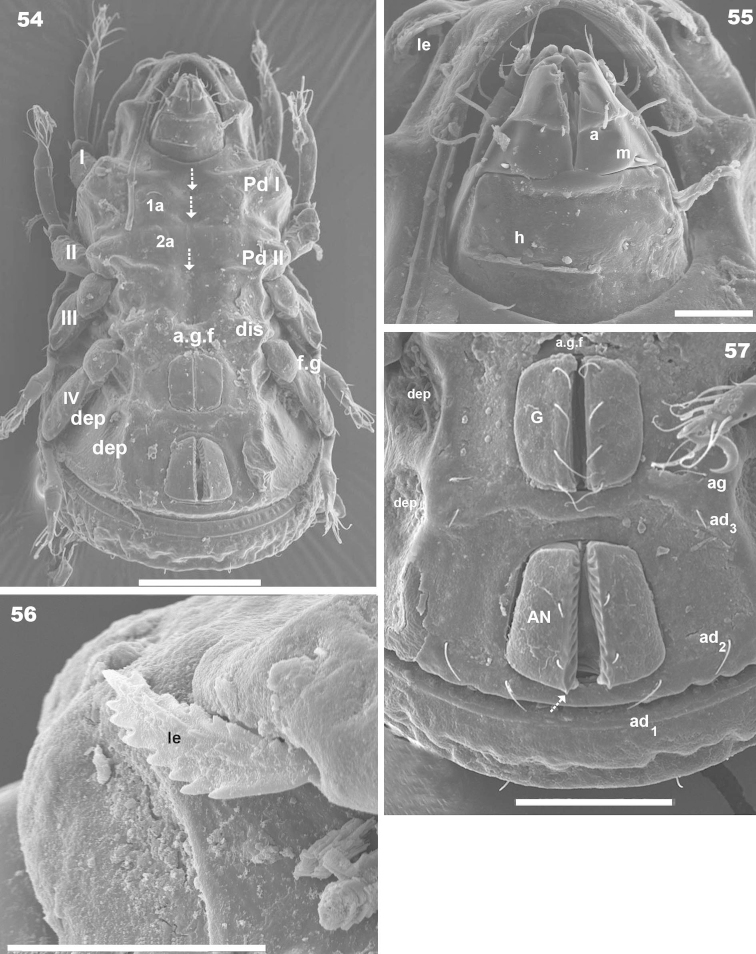
*Machadocepheus rachii* sp. n., adult female. SEM observations. **54** ventral view (2) **55** subcapitulum, ventral view (1); **56** genito-anal zone (1) **57** lamellar tip (2). Abbreviations: see “Material and methods”. Scale bar: **54** = 100 μm; **55, 56** = 20 μm; **57** = 50 μm.

**Figures 58–63.**
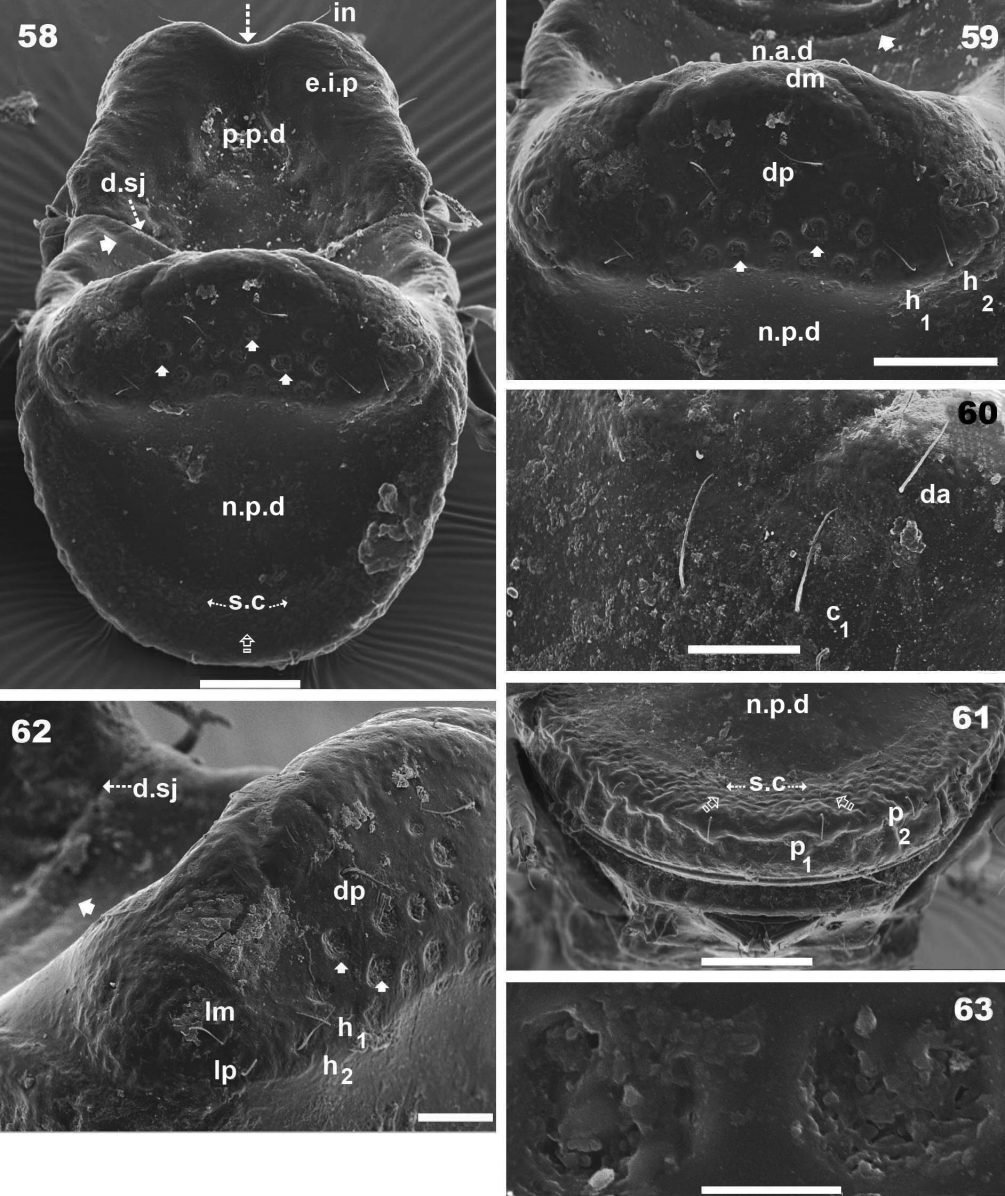
*Machadocepheus rachii* sp. n., adult female. SEM observations. **58** posterior view (2) **59** notogastral elevated zone; dorsoposterior view (2) **60** notogaster, zone insertion *c* and *d* (2) **61** notogastral posterior zone; posterior view (2) **62** notogastral elevated zone, posterolateral view (2) **63** notogastral ornamentations (2). Abbreviations: see “Material and methods”. Scale bar: **58, 59, 61** = 50 μm; **60, 62** = 20 μm; **63** = 5 μm.

***Prodorsum*.** Polyhedral (dorsal view) (Figures 38, 41); triangular (lateral view) with strong obliquely decreasing anterior part (Figures 40, 46, 47). Interlamellar process (*e.i.p*) elevated (Figures 40, 46, 47), divided sagittally into two promontories by large deep furrow (Figures 41, 42, 47, 58 indicated by ⇣), *in* setae situated anteriorly and directing posteriorly (Figures 42). Conspicuously depressed posterior prodorsal zone (*p.p.d*) (Figures 38, 41, 47, 58). Three pairs of setae; sizes *in*> *le*> *ro. Ro* setae clearly visible in frontal view (Figures 42, 44), situated in medial zone, inserted posterior to insertion level of *le*; rounded structure between *ro* setae, probably vestigial superior cornea of naso *cso* (Figures 42, 44); *bo* cup-shaped, *bo.ri* smooth; bothdial tooth present (Figures 46, 51). *Si* uncinate, upturned (Figures 46, 51); *le* setae situated ventrally on apical zone of lamellae (Figures 42, 44, 56).

Rostral margin slightly rectangular to hexagonal (Figures 42, 44). Lamellae running dorso laterally, lacking lamellar tip (Figures 42, 44, 56); large deep furrow (*l.l.f*) demarcating inner paraxial margin of lamellae (Figures 41, 42, 47). In frontal view (Figure 42), *l.l.f* showing a deeper medial zone. The superior cornea of naso (*cso*) clearly visible as convex elevation situated anterior to *ro* setal insertion level (Figures 42, 44).

***Notogaster*.** Shape: dorsal view, anterior part rectangular and posterior part oval (Figures 38, 41, 58); in lateral view, anterior part rectilinear, with clearly concave medial zone and rectilinear exteriorly, rest triangular to polyhedral with some irregularities and unremarkable promontories (Figures 40, 46, 47, 48, 53); *d.sj* narrow, rectilinear, hardly discernible (Figures 41, 47); notogastral anterior depression (*n.a.d*), ovoid and conspicuous.

Fifteen pairs (holotrichy unideficient) of notogastral setae: *c_1_*, *c_2_*, *c_3_*, *da*, *dm*, *dp*, *la*, *lm*, *lp*, *h_1_*, *h_2_*, *h_3_*, *p_1_*, *p_2_*, *p_3_*.

Notogaster presenting: 1) *n.a.d* occupying anterior notogastral zone; 2) elevated zone situated in posterior third of notogaster; 3) slightly concave *n.p.d* situated posterior to elevated zone (Figures 41, 46, 47, 48); 4) slightly inclined more or less flat zone situated behind *s.c*, with irregularly elongated protuberances (Figures 41, 58, 61 indicated by ⇪); circumgastric depression (*s.c*) hardly discernible (Figures 53, 58, 61).

Simple *n.a.d* (Figures 38, 41, 47) with many hardly discernible transversally aligned parallel cuticular folds situated posterior to *d.sj* (Figures 47, 58, 59, 62 indicated by ➧). Elevated zone presenting series of aligned flat medial promontories (three pairs, poorly developed) bearing setae *da*, *dm*, *dp* and lateral poorly developed semicircular promontories, bearing setae *la*, *lm*, *lp*, *h_1_*, *h_2_*.

Humeral apophysis (*h.ap*) very long, clearly visible (Figures 38, 41) but best observed in lateral view (Figures 46, 47).

***Lateral region*** (Figures 40, 46, 47, 48). Palp clearly discernible (Figure 50), *sul* ζ (*ul* ζ), *acm* ζ; solenidium ω very long, extending to level of eupathidia. Cheliceral setae *chb* clearly visible (Figure 50).

Lamellae (*lam*) easily discernible, large, lacking sharp *la.ti*, with elevated zone at *le* insertion level (Figure 40, 46, 47, 56).

Tutorium (*tu*): rod-like curving ridge; *s.tu.d* a deep depression running between lamellae and tutorium; pocket depressions *a.tu.d*, *p.tu.d* present; another small depression situated internally to *s.tu.d* (Figure 40 indicated by ⇪).

Bothridia cup-shaped, *bo.ri* incomplete, *bo.to* present, clearly discernible (Figures, 46, 47, 51). Sensillus uncinate, arched, curving upward (Figure 45, 46, 47). Pd I: prominent extended lamina, rounded apex; Pd II: small ovoid lamina (Figures 40, 46, 47); *h.ap* long extending structure, rounded apex, basally curved; anterior tip overlapping posterior bothridial posterior part (Figures 40, 46, 47).

Notogastral promontories and setae very clearly discernible (Figures 47, 52, 53).

Only lyrifissures *ih* and *ips* clearly visible. Discidium easily discernible as polyhedral structure with rounded apex. Several depressions (*dep*) clearly discernible behind acetabulum IV; one of them elongated, concealing the tarsus during leg folding process (Figure 40).

***Ventral region*.** Infracapitulum with setae *h*, *m*, *a* clearly visible (Figures 39, 55). Epimeres slightly elevated, delimited by shallow furrow (*bo.1*, *bo.2*, *bo.sj*). In medial zone a series of aligned depressions (Figure 54 indicated by ⇣); Apodemes (*apo.1*, *apo.2*, *apo.sj*, *apo.3*) well discernible (Figures 39). Epimeral chaetotaxy 3–1-3–3; *Pd I*, *Pd II* and *dis* well discernible; *a.g.f* clearly visible, situated anteriorly to genital fig (Figures 39, 54). Genital fig small relative to anal fig (Figures 54, 57); four pairs of long genital setae (Figure 57); anal fig with two pairs of small but clearly discernible setae; fig terminating in small sharp tip (Figure 57, indicated by ⇡); *ag* and *ad_3_* equal in length; *ad_2_* and *ad_1_* situated laterally at level of posterior end of anal fig (Figure 57). Lyrifissure *iad* not discernible. Particular depression behind acetabulum IV (Figure 39 indicated by ↖). Several large depressions laterally to anal and genital figs and marginally to ventral shield (Figures 39, 54, 57).

***Posterior view*.** This view permits clarification of several aspects such as: a) shape of the *e.i.p* and large depression in the anterior medial zone (Figure 58 indicated by ↓); b) shape and depth of *p.p.d* (Figure 58); c) shape and disposition of *d.sj* (Figure 58, 62 indicated by ⇣); d) disposition of the transversal cuticular folds situated behind *d.sj* (Figures 58, 59, 62, indicated by ➧); e) disposition, shape and distribution of setae and cuticular ornamentations on elevated notogastral zone (Figures 58, 59, 62). f) disposition of *sc* and the zone with irregularly elongated protuberances (Figures 58, 61 indicated by ⇪).

***Legs*** (Figures 64–72). All legs monodactyle. Setal formulae I (1-4-2-4-16-1) (1-2-2) (Figure 67); II (1-4-3-2-15-1) (1-1-2) (Figure 64) III (2-3-1-2-13-1) (1-1-0) (Figure 70); IV (1-2-2-2-12-1) (0-1-0) (Figure 69).

**Figures 64–72.**
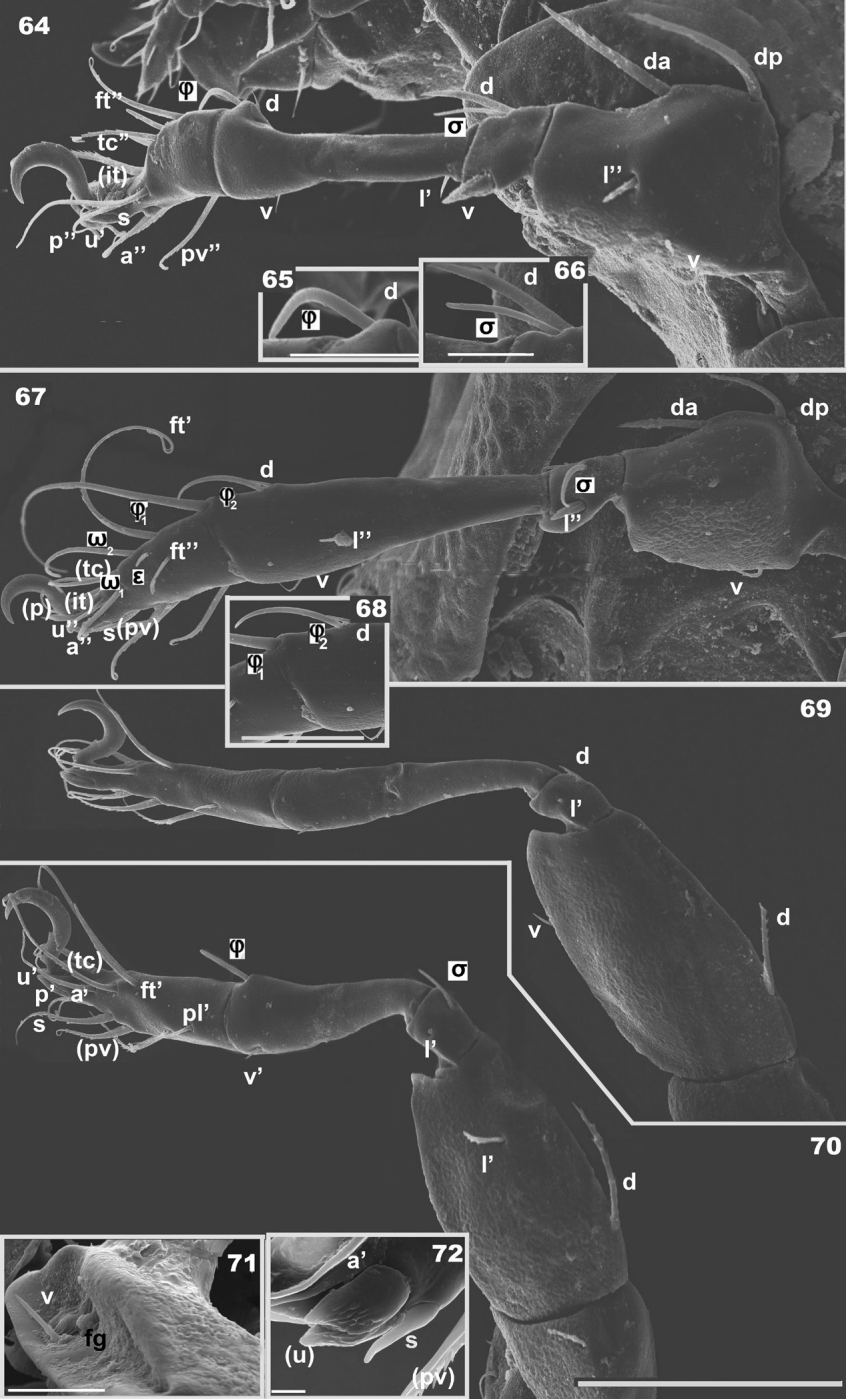
*Machadocepheus rachii* sp. n., adult female. SEM observations. **64** leg II, antiaxial view (1) **65** solenidion φ and dorsal setae of Tibia II (1) **66** solenidion σ and dorsal seta of genu II (1) **67** leg I, antiaxial view (1) **68** solenidion φ_2_ and dorsal setae of Tibia II (1) **69** leg IV, antiaxial view(1) **70** leg III antiaxial view (1) **71** femoral groove, femur leg III (2) **72** apical zone, tarsus III (1). Abbreviations: see “Material and methods”. Scale bars: **64, 67, 69, 70** = 50 μm; **65, 66, 71** = 10 μm; **68** = 20 μm, **72** = 2 μm.

Seta *d* of tibia I associated with φ_2_ (Figure 68). Setae *d* on tibia II small (Figure 65), situated behind φ, not associated with solenidion; setae *d*, genu II (Figure 66), large, situated behind and associated with σ. Femur III with femoral groove *f.g*, difficult to observe in antiaxial view (Figure 70), but well developed, containing seta *v* (Figure 71); disposition of setae on tarsus III: (*u*), *s*, (*a*) (Figure 72) particular *s* situated anterior to (*a*). Femur IV presenting a conspicuous ventral carina (Figure 69).

## Discussion

Intricate structural shapes and the need to observe specimens from various angles and positions made many structures difficult to understand when only using optical observation. Comparing these species with others from the same genus was greatly complicated by very short and superficial original descriptions, and some errors were detected in descriptions of various species of the genus *Machadocepheus* as well as in related genera (*Bathocepheus*, see Fernandez et al. 2013; *Tuberocepheus* see Fernandez et al. 2014). Much care had to be taken not to create any further confusion in the genus *Machadocepheus* and related genera, and for the reasons cited above we deemed it necessary to continue our study of a number of related genera in a series, discussed in future papers, to try to understand the existing problems.

## Supplementary Material

XML Treatment for Machadocepheus leoneae

XML Treatment for Machadocepheus rachii
